# Characterization of talc deposits in ultramafic rocks of Gebel El Maiyit and its economic feasibility

**DOI:** 10.1038/s41598-025-97465-7

**Published:** 2025-04-24

**Authors:** Ibrahim A. Salem, Samir M. Aly, Ali Shebl, Ismail A. Thabet

**Affiliations:** 1 Department of Geology, Tanta University, Tanta, 31527 Egypt; 2 Department of Mineralogy and Geology, University of Debrecen, Debrecen, 4032 Hungary

**Keywords:** Ultramafic rocks, Talc deposits, Remote sensing, Stable isotope analysis, Eastern desert, Egypt, Whole rock chemistry, Geochemistry, Mineralogy, Petrology

## Abstract

A comprehensive, multiscale investigation, integrating remote sensing, mineralogy, whole rock chemistry, Electron Microprobe (EMP), and stable isotopes (oxygen-^18^O and carbon-^13^C), was undertaken to assess the feasibility of talc deposits and their host serpentinite at Gebel El-Maiyit in the Eastern Desert of Egypt. Sentinel 2 remote sensing images were applied to discriminate talc from serpentinites followed by geochemical study of serpentinites using RO`/SiO_2_ ratios, AFM diagram and MgO versus SiO_2_ relationship indicates a peridotite origin formed at low temperature Alpine type. Our study revealed that talc deposit has a varied mineralogical composition and according to the predominant talc and gangue minerals three main types have been distinguished: 1- pure talc, 2- tremolite talc and 3- chlorite talc. Paragenetically, talc is derived from serpentine minerals, tremolite and chlorite. The latter is formed at about 231 °C. The chemical data of talc deposit reveals that the summation of talc components (SiO_2_ + MgO + H_2_O) is 92.68%, while that of impurity oxides (Al_2_O_3_ + CaO + Fe_2_O_3_ + FeO) is 5.56%. The carbon^13^C) and oxygen^18^O) contents of pure magnesite revealed that the pure phase of Gebel El-Maiyit was formed at low temperature (around 100 °C) while magnesite contained in talc carbonate rock was formed at high temperature (140–175 °C). In terms of source fluids, the metamorphic and /or magmatic water was supposed to be the main fluids which are circulated during the hydrothermal alteration. Although S and P are very minor components in all the talc ore types of the considered area and do not affect their industrial use. Copper (Cu) was not detected. Iron (Fe) and manganese (Mn) concentrations are significantly high, necessitating treatment to reduce these elements for the ore to be suitable as an electrical insulator. Arsenic (As) levels are consistently below 5 ppm, indicating the ore’s potential use in the cosmetic industry without further processing.

## Introduction

The Arabian-Nubian Shield (ANS) originated during the Neoproterozoic, coincident with the closure of the Mozambican Ocean^1–3^. During the collision the Mozambique oceanic crust subducted beneath several oceanic arcs and influenced by recycling of water and volatile rich fluids that led to melting, metasomatism and serpentinization^4,5^. The progressive characterization of serpentinites, from the Eastern Desert of Egypt terms of their tectonic setting, show their magmatic processes and tracing the mantle evolution, the nature of the mantle melting and the enrichment processes during the Neoproterozoic time was studied by Ref^6^. The ultramafic rock, island metasediments and granitic rocks are encountered in Gebel El-Maiyit province, on ultramafic rocks and various forms hydrothermal alteration contain the essential type as serpentinites and talc carbonates rocks.

Talc deposits occur on every continent and the geology of these deposits is roughly similar to those of Egypt. The metamorphic environment is most common area of formation which largely confined to the Precambrian age. Talc deposits can be divided into four types according to their assumed origin as reported in much literature^7–12^. The quality and economics of each deposit are largely controlled by its origin. Talc deposits can be formed in ultrabasic and serpentinized rocks, mafic igneous rocks (gabbro, and basalt), andesite, metasomatic (hydrothermally) altered dolomite and dolomitic limestone (metasedimentary origin), regionally or contact metamorphosed siliceous to sandy dolomite or siliceous talc-carbonate.

Talc deposits occurring in the form of lenses, veins and discontinuous narrow bands mostly with low quality talc originate from ultramafic rocks. The thicker and purer talc deposits originate from metamorphosed dolomite. Commercially, these are the most important. Talc deposits are found in many countries in the world. The large consumers of talc are America, Europe and Japan. Talc can be formed under laboratory conditions at temperature range of 500-700^o^C is reported by Kuvart^11^ the experiments have shown that, in nature talc originates from true and colloidal solutions through reaction between magnesia and silica in hydrothermal environment in the later stages of contact or regional metamorphism (greenschist to amphibolite). Talc {Mg_3_Si_4_O_10_(OH)_2_} is hydrous magnesium silicate found as a soft crystalline mineral (foliated or non-foliated) of white, green or grey color and soapy feel. Talc is secondary mineral formed due alteration product of the original or other secondary Mg-rich minerals by hydrothermal solution or hot magmatic water. The parent minerals include tremolite, chlorite, olivine, serpentine, dolomite, magnesite and enstatite.

Talc has a wide range of uses in industry. Talc is classified industrially into hard and soft, friable and flaky and is marketed as crude, ground and sawed. The industrial application depends on its properties such as color, flakiness, powdered density, chemical inertness, oil absorption value and presence or absence of impurity materials. For many industrial applications, a wide variety of qualities are required, ranging from very pure white talc to grey colored talc perhaps containing as much as 50% impurities. The main uses of talc are: paint extender, cosmetics, steel marking, pharmaceutical, polish for rice, wood, nails, white shoes, ceramics base materials for wall and floor tile as well as filler in the plastic, paper, roofing and rubber industry.

Talc is found in many localities in the Eastern Desert of Egypt where it is associated principally with host metavolcanic and serpentinite rocks. The geology, mineralogy, chemistry and origin of the Egyptian talc deposits hosted by serpentinite rocks were studied by Basta and Kamel^13^, Hussein^14^, Salem^15^, El-Sharkawy^16^. The Neoproterozoic ophiolites in the Arabian-Nubian Shield (ANS) age vary from 690 to 890 million years ago and up to 100 Ma (from 780 to 680 Ma) of terrane development in suture zones^1,17–19^.

**Fig. 1:**
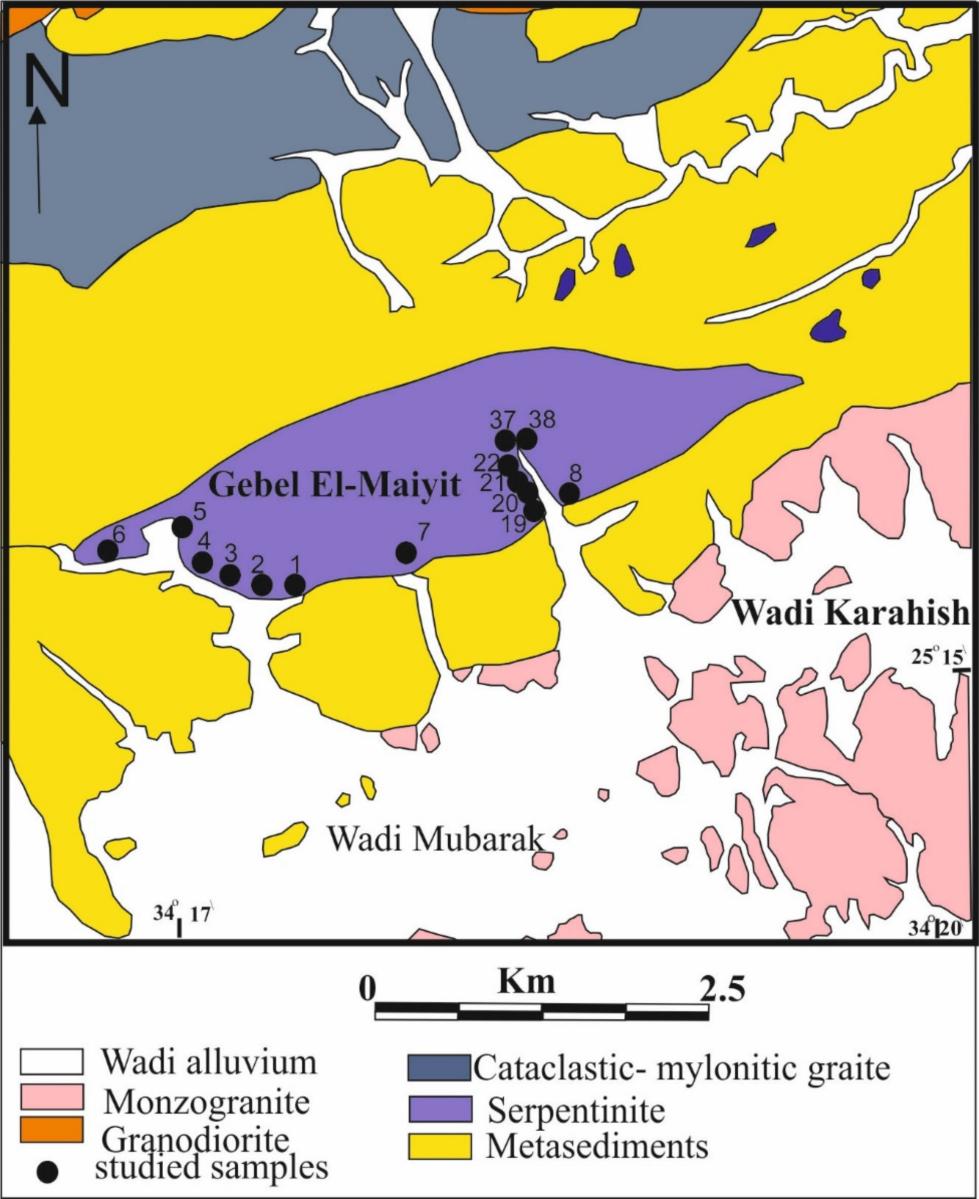
Geological map of Gebel El-Maiyit (after Abu El-Ela 1985^34^).

Remote sensing techniques have proven to be effective tools for mapping and studying the distribution of different rock types^20–22^. In particular, the use of multispectral satellite imagery has allowed researchers to identify and locate specific minerals and lithological units^23,24^. This study aims to detect and map the distribution of talc deposits associated with ultramafic of Gabel El Maiyit (Fig. 1).

The present work is primarily concerned with discussing the geological setting, mineralogy and petrochemistry of the talc deposits hosted by serpentinite rocks of Gebel El-Maiyit and to present an explanation for the genesis of the talc deposits.

## Geologic setting

The Neoproterozoic rocks of Egypt straddle the northern sector of the Arabian Nubian Shield (ANS), which represents the northern extension of the highly deformed Mozambique Belt. ANS makes up the eastern limb of the U-shaped Pan African Orogenic belt, one of the best examples of juvenile continental crust in the world^26–31^. The ANS encompasses both sides of the Red Sea: the Nubian Shield (western sector) and the Arabian Shield (Eastern sector)^31–33^.

**Fig. 2:**
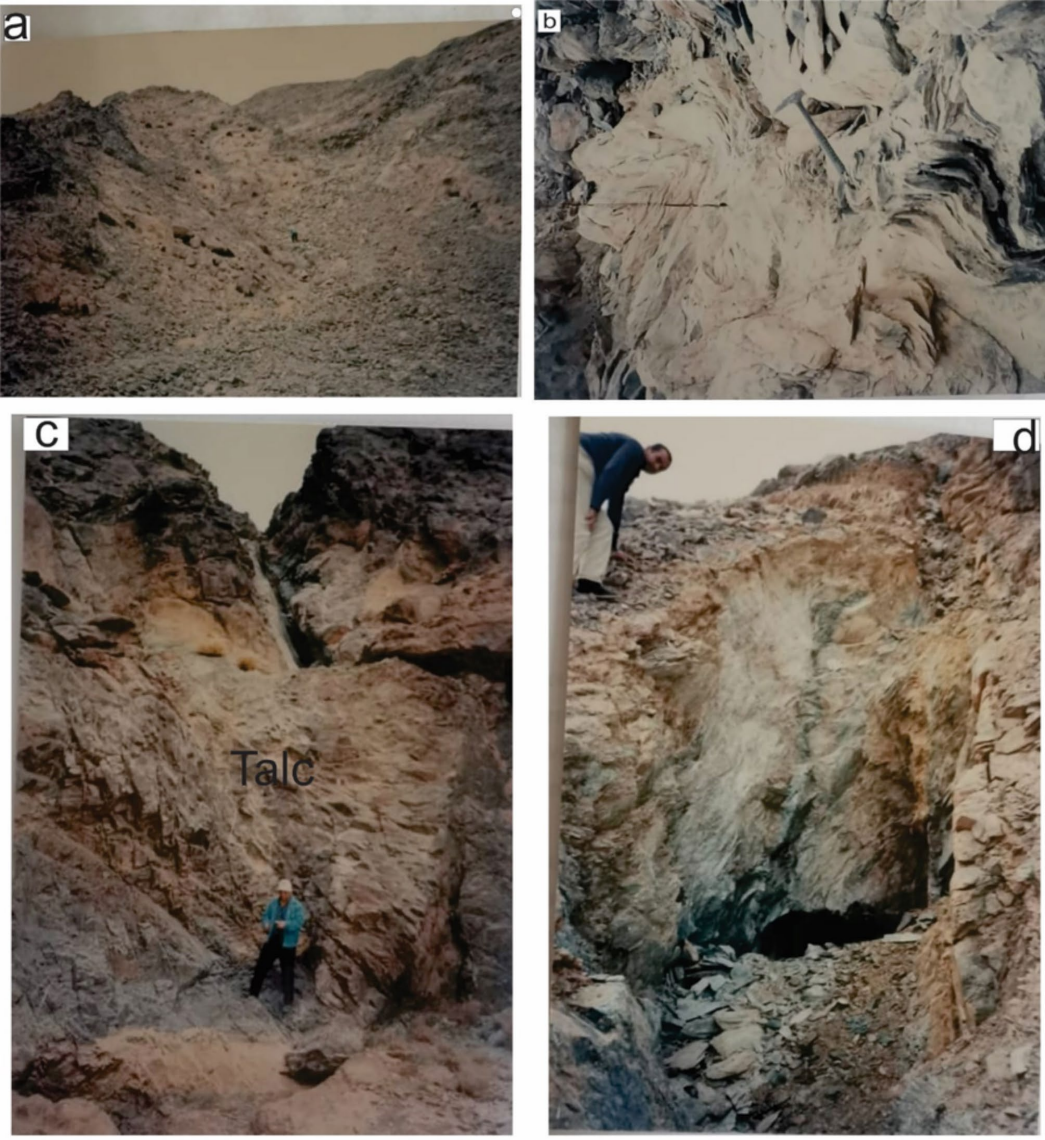
(**a**) Talc carbonate rock capping serpentinite showing cavernous structure. (**b**) close up view small folding in talc flakes as surrouding by serpentinite rocks. (**c**) close up view show asheet like body wihich is wholly mined in same extension of dyke. (**d**) close up view showing underground opening within serpentinite body for talc mining. These field photographs are taken by the authors of the current research. These photos are our own and we agreed to publish them.

Gebel El-Maiyit talc deposit lies in the central Eastern Desert (CED) of Egypt, at the intersection of latitude 25° 16’ 00” and longitude 34° 18’ 30”. It is located to the North of Idfu-Mersa Alam road and about 85 km from Mersa Alam on the Red Sea coast. The ultramafic rock of G. El-Maiyit is considered one of the largest ultramafic bodies in the CED and belonging to ophiolite suite of the ED of Egypt^34,35^. The serpentinite mass forms an elongated body of lenticular outcrop extending 6–7 km in length in a ENE-WSW direction. It has a maximum outcrop width of 1.6 km and more than 200 m vertically is visible (total area extent of 7 km², Fig. 1). The host rock is made mostly of completely serpentinized ultramafic rocks of alpine type emplaced in folded metasedimentary and metavolcanic rocks. The serpentinite rocks which host talc are made essentially of dense, dark green and homogeneous serpentinite. Neither fresh dunite nor peridotite rock is encountered. The parent ultramafic pluton of G. El-Maiyit has undergone extensive alteration (serpentinization). It shows various degrees of alteration of talc and talc- carbonate Fig. 2.

## Material and analytical techniques

Several techniques have been used in research including, sample collection, petrography, whole rock chemistry, mineral chemistry and isotopes. The work has been done at the laboratories of Camborne School of Mines, University of Exeter, UK. Rare earth elements (REE) were analyzed at Greenwich University while carbon and oxygen isotopes at Liverpool University.

### Remote sensing data

The acquisition of cloud-free Sentinel 2 A data was done through the European Space Agency (ESA) to fulfill the objectives of the present research. Table 1 outlines the spectral and pixel-size characteristics of the Sentinel 2 data^36^. Subsequently, the sen2cor processor, integrated within the Sentinel Application Platform (SNAP), was utilized to preprocess these datasets, primarily employing radiometric methods. Following preprocessing, the data was further processed and adjusted to align with the boundaries of the study area of Gabel El-Maiyit area, is known for diverse geological formations, making it an ideal location for investigating the effectiveness of remote sensing techniques in identifying and characterizing Talc deposits associated ultramafic. To enhance the spatial resolution of the Sentinel 2 data, information from the Panchromatic Remote-sensing Instrument for Stereo Mapping (PRISM) was incorporated. PRISM, hosted by the renowned ALOS (Advanced Land Observing Satellite), specifically contributed to digital elevation mapping with a pixel size of up to 2.5 m^37^ and its characteristics are shown in Table 1.

**Fig. 3:**
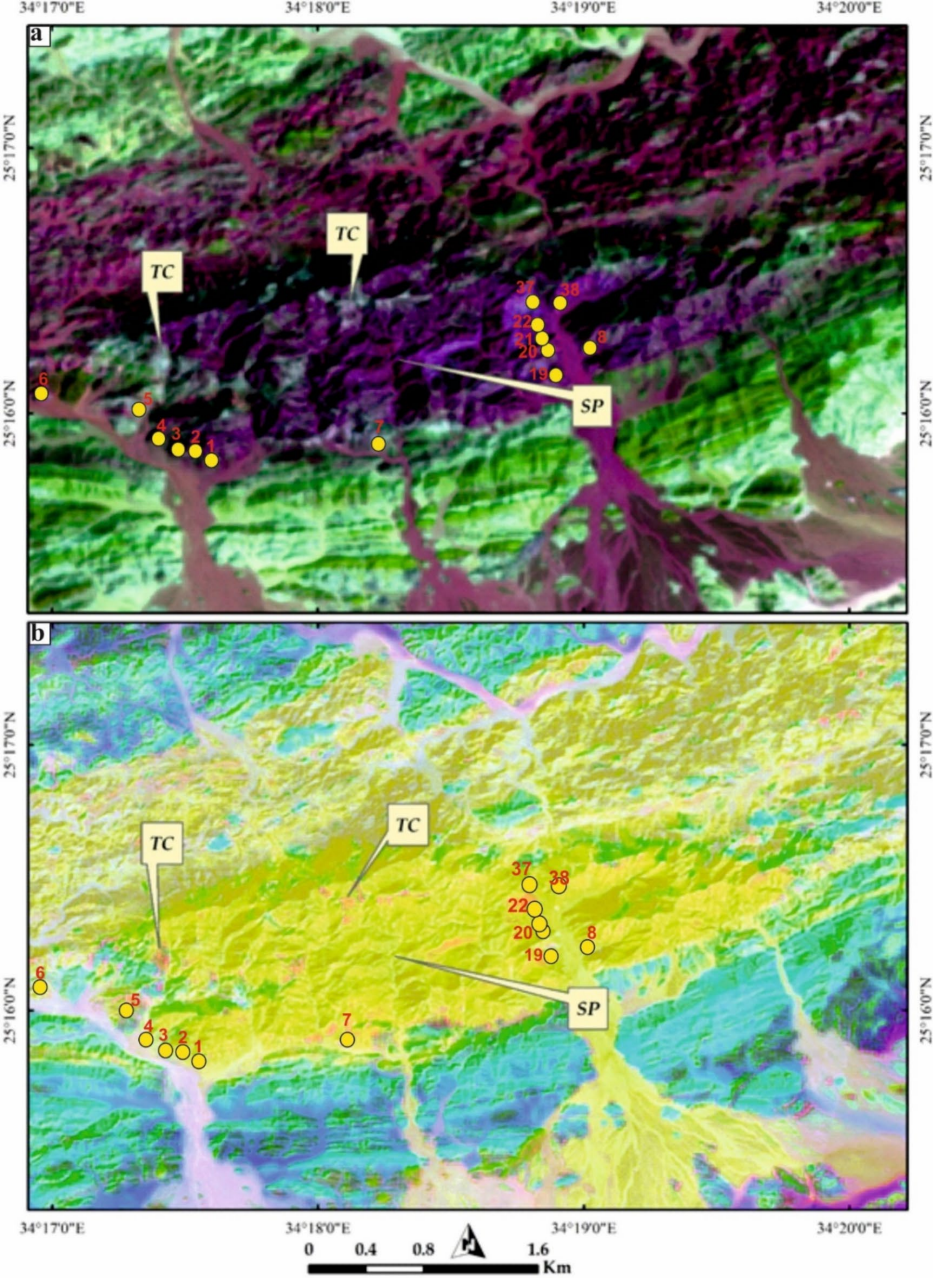
Lithological discrimination of Gebel El-Maiyit serpentinite (central part, approximately sigmoidal shape) utilizing 10 m pixel size sentinel 2 combinations of (**a**) FCC 2-12-6 in RGB and (**b**) PC2-PC1-PC3 in RGB, respectively.

The primary objective of utilizing remote sensing data is to distinguish talc-carbonate rocks from their host, serpentinite rocks Fig. 3. To achieve this goal, numerous image combinations were generated, and diverse image processing methods were employed. However, our study found that false color combinations (FCC), principal component analysis (PCA), and band ratios yielded the most effective results. Although FCC (False Color Composite) is considered traditional in remote sensing, its three RGB bands still offer utility across a broad spectrum of applications. The choice of bands is primarily determined by the specific features being analyzed. For instance, because iron-rich minerals exhibit distinctive absorption properties within VNIR (Visible and Near Infrared) spectral range, VNIR bands are commonly integrated into geological remote sensing systems for their differentiation. SWIR (Shortwave Infrared) bands are preferred for highlighting carbonates and minerals containing OH groups. Given the significant compositional diversity of the exposed rock units in the current study, the most effective image composite for distinguishing these units was created by presenting Sentinel 2 band 12 (SWIR) in red, band 6 (VNIR) in green, and band 2 (blue) in blue.

As stated by Crosta. 1989^38^, Moore. 1981^39^, Loughlin. 1991^40^, Abdelkader et al. 2022^41^; Shebl and Hamdy, 2023^37^, Principal Component Analysis (PCA) is a statistical method that generates new components (PCs) from the original dataset. This transformation often reveals new characteristics and enhances discrimination, particularly when combined with previously informative components. The band ratio technique involves dividing the digital number (DN) values of one band by those of another band. The resulting DN values are then represented as a grayscale image, providing relative band intensities, as described by Ref^42^.

**Table 1:** Characteristics of Sentinel 2 and PRISM datasets^36^.

Sentinel 2			
Band	Spectral region	Central wavelength (µm)	Spatial resolution (m)
B1	Ultra blue	0.443	60
B2	Blue	0.490	10
B3	Green	0.560	10
B4	Red	0.665	10
B5	VNIR	0.704	20
B6	VNIR	0.740	20
B7	VNIR	0.782	20
B8	VNIR	0.842	10
B8a	VNIR narrow	0.865	20
B9	SWIR water vapor	0.945	60
B10	SWIR cirrus	1.375	60
B11	SWIR	1.610	20
B12	SWIR	2.190	20
PRISM			
Number of Bands		1 (Panchromatic)	
Wavelength		0.52 to 0.77 micrometers	
Number of Optics		3 (Nadir; Forward; Backward)	
Base-to-Height ratio		1.0 (between Forward and Backward view)	
Spatial Resolution		2.5 m (at Nadir)	
Swath Width		70 km (Nadir only) / 35 km (Triplet mode)	
S/N		> 70	
MTF		> 0.2	
Number of Detectors		28,000/band (Swath Width 70 km) 14,000/band (Swath Width 35 km)	
Pointing Angle		−1.5 to + 1.5 degrees(Triplet Mode, Cross-track direction)	
Bit Length		8 bits	

### Sampling and petrological and XRD examinations

More than thirty samples were collected from G. El-Maiyit, their mineralogical composition determined by polarizing microscope, reflected microscope for opaques minerals and XRD investigation for 10 samples of serpentinite rocks, 3 samples of talc carbonates rocks and 9 from talc ore.

### Petrochemistry and mineral chemistry of serpentinite rocks

Thirteen samples from serpentinite and associated talc-carbonate were chemically analyzed. Major and trace elements were analyzed by using Phillips PW 1400 X-ray fluorescence, XRF. Only four samples were subjected to the REE analysis by ICP-MS. Four samples analyzed for REE by Inductively Coupled Plasma Spectrometer (ICP-MS).

### Oxygen and carbon isotopes

The carbon and oxygen isotope study was performed of 4 carbonate-rich samples. On the basis of petrographic studies (cathodoluminescence and stained uncovered thin sections), samples containing different types of carbonate minerals were selectively chosen. Results are expressed in standard as per mil notation relative to Pee Dee Belemnite (PDB) and standard mean oceanic water (SMOW). Analytical error is in the range of 0.1–0.2 per mil for both^18^O and^13^C. The present analyses were carried out at the University of Liverpool Stable Isotope laboratory.

## Results and discussion

### Remote sensing processing

Our remote sensing spectral discrimination was evident, depicting Gebel El-Maiyit serpentinite with a dark grey-black hue in FCC 2-12-6 (RGB) as shown in Fig. 3a. Our data proved to be useful in clearly discriminating Gebel El-Maiyit serpentinite from other lithologies, including exposed metasediments and granitic rocks, using Sentinel 2 data. El-Maiyit serpentinite was distinctly identified, depicted as an approximately sigmoidal lense trending mainly NE-SW (Figs. 3a and b). Furthermore, better discrimination of these serpentinite rocks was observed using the PC2-PC1-PC3 combination in RGB after enhancing the contrast, reflecting our lithological target as massive-deep yellow, distinguished from the surrounding areas (Fig. 3b).

**Fig. 4:**
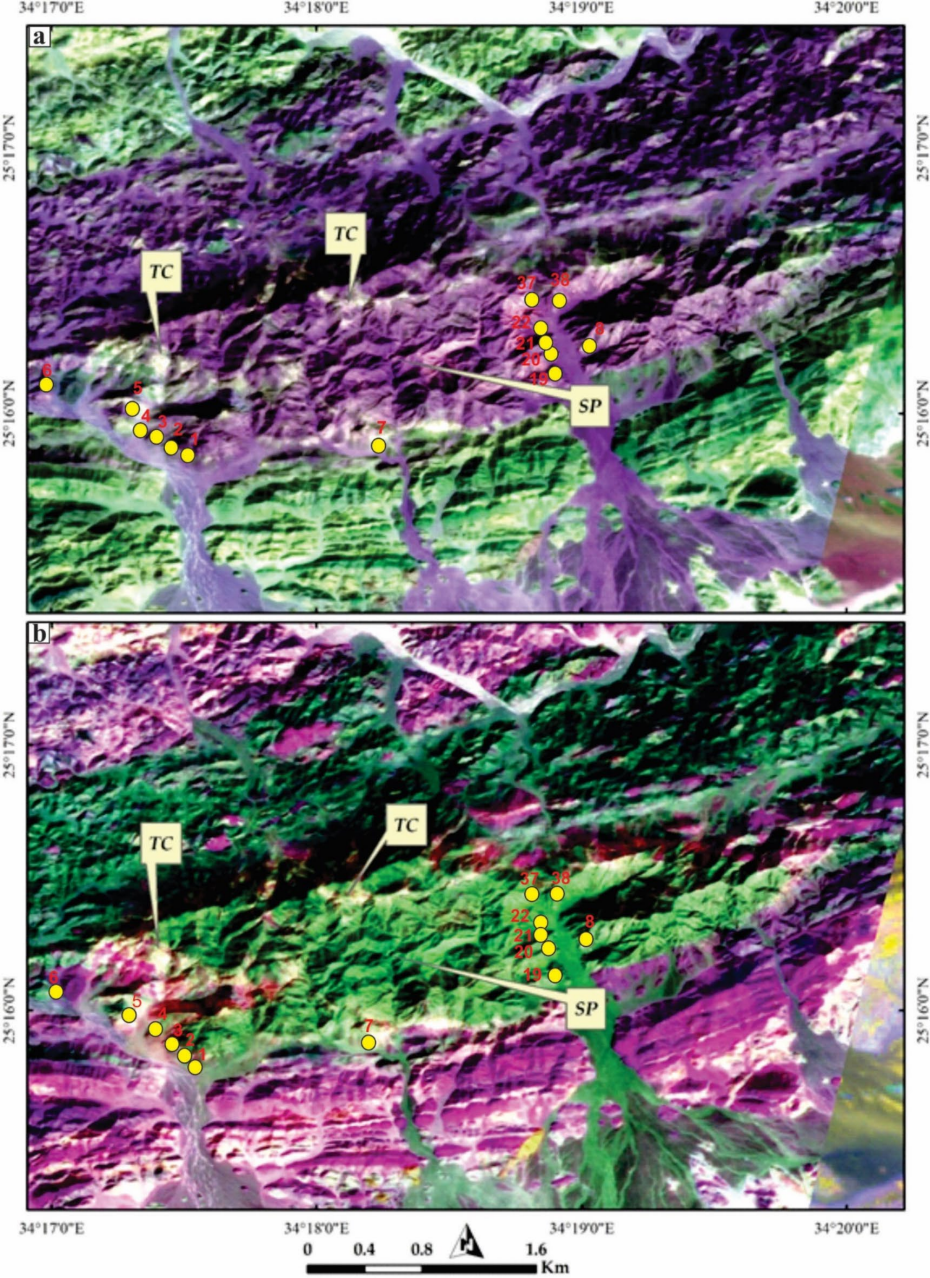
Detailed (2.5 m) mapping of talc-carbonates (Tc) associated with Gebel El-Maiyit serpentinite (SP) using PRISM pan-sharpened Sentinel 2 combinations of (**a**) FCC 2-12-6 in RGB and (**b**) BR11/7–8/2–12/5 in RGB, respectively. These photos are our own and we agreed to publish them.

However, our primary challenge was not only discriminating the host rock serpentinites but also depicting the distribution of their associated talc carbonates. Therefore, we created PRISM pan-sharpened Sentinel 2 combinations (2.5 m) to enhance the spatial separability of these rock bodies. Our results revealed unambiguous discrimination of these talc carbonates (Tc), as shown in Fig. 4. These rocks are primarily distributed along the borders of Gebel El-Maiyit serpentinite. Figure 4a clearly highlights them as light pixels, almost associated with the serpentinite rocks. These pixels are further confirmed in Fig. 4b as bright, revealing how the spatial enhancement using PRISM aids the current research in better delineating their distribution for further analysis.

### Petrography and geochemistry of the serpentinite hosted rocks

**Fig. 5:**
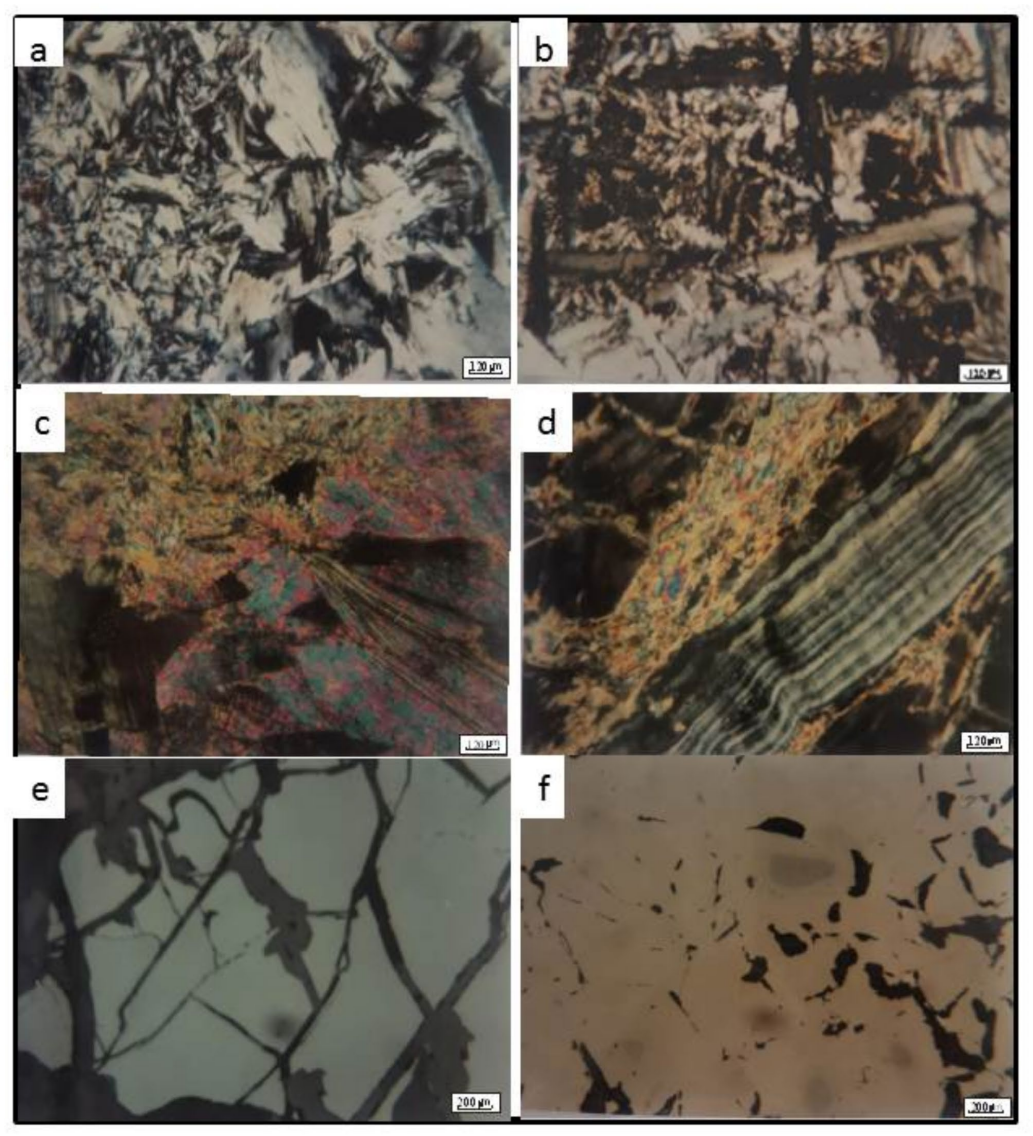
(**a**) Two forms of antigorite the fine anhedral plates (formed first) are in contact with the coarse tabular ones (formed later). (**b**) Antigorite blades in perpendicular arrangement reflecting the pyroxene cleavage planes. (**c**) Alteration of chlorite to talc with remnants from cleavage planes and crystal form in matrix of talc. (**d**) Vein of chrysotile in a matrix of talc in the chlorite talc ore type. (**e**) The fractures in chromite, which may be developed due to the tectonic effects during serpentinization process. (**f**) Replacement of chromite by ferrochromite is developed until only remnants form chromite is preserved.

The mineral assemblages of serpentinite and talc carbonate were determined and confirmed by both microscopic and XRD investigations Fig. 5 and listed in (Table 2). Three main varieties of serpentine minerals are recognized; antigorite, lizardite and chrysotile. Antigorite shows great abundance relative to lizardite and chrysotile. Magnesite and dolomite are also found as accessory constituents of serpentine. Chromite and magnetite are abundant and are found as a mass or as fine grains outlining the original boundaries of the parent minerals.

**Table 2:** The result of XRD of the mineral assemblage of serpentinite and Talc carbonate rocks.

Rock type	No	Major	Minor	Rare
Serpentinite Rocks	22	Antigorite	Lizardite	Talc, Chromite
	21	Antigorite	Lizardite	Talc, Chromite
	20	Antigorite	Lizardite	Dolomite, Chromite
	3	Antigorite	Lizardite	Magnesite, Dolomite, Chromite
	37	Antigorite	Magnesite, Lizardite	Dolomite, Chromite
	38	Antigorite	Lizardite	Magnesite, Dolomite, Chromite
	1	Antigorite	Lizardite	Dolomite, Chromite
	4	Antigorite	Lizardite	Magnesite, Dolomite, Talc Chromite
	6	Antigorite	Lizardite, Dolomite	-
	19	Antigorite	Lizardite	Dolomite, Chromite
Talc carbonate Rocks	8	Talc	Magnesite	Chlorite, Antigorite, Chromite
	7	Magnesite	Talc	Chlorite, Lizardite, Kaolinite, Calcite Chromite
	5	Talc	Dolomite	Chlorite, Antigorite, Kaolinite

### Petrochemistry of serpentinite rocks

The results of major and trace elements analyses, loss on ignition (LOI), the normative mineralogy and calculated amount of H2O are presented in Table 3.

**Fig. 6:**
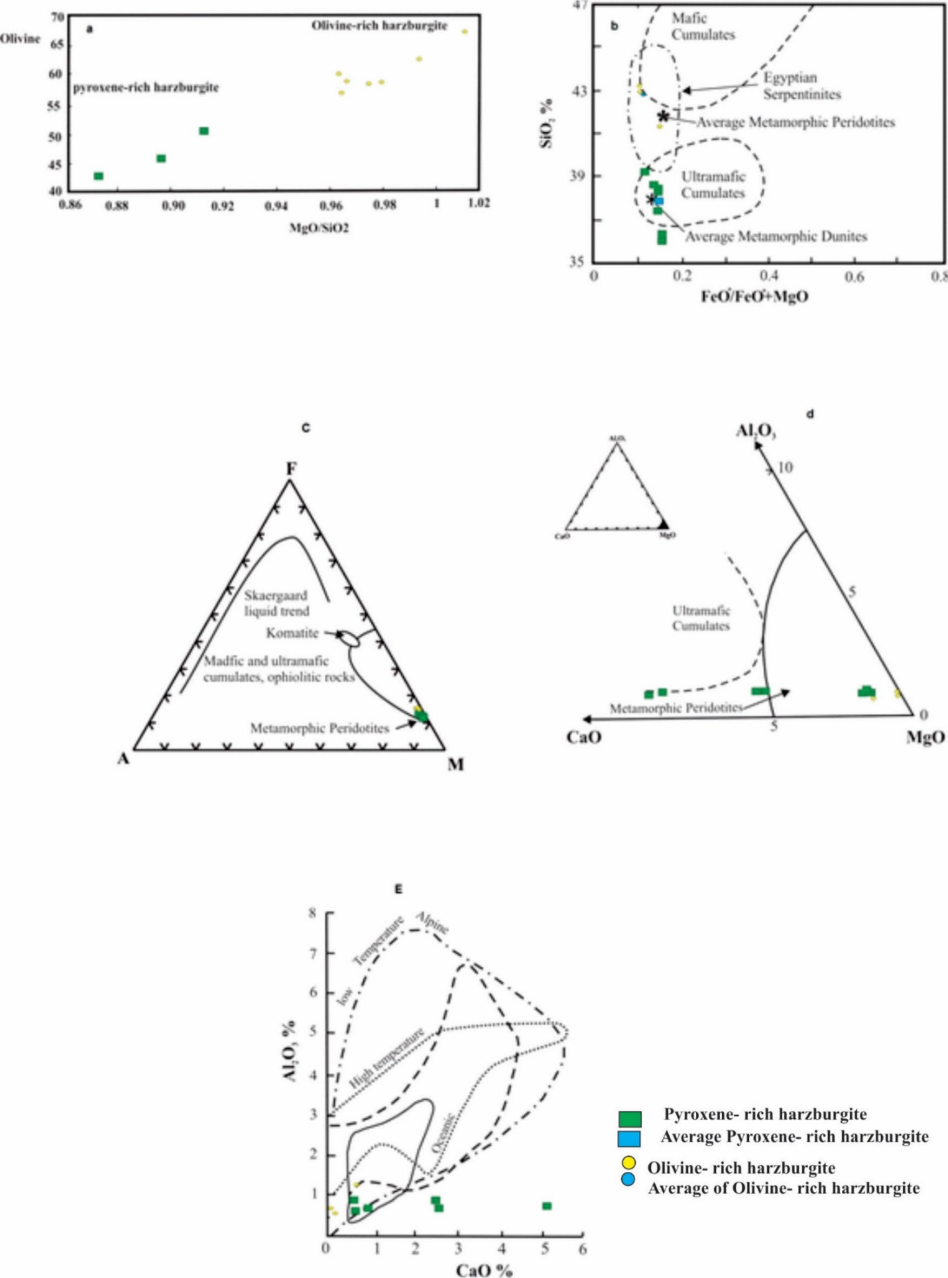
(**a**) The relation between olivine content and MgO/SiO_2_ (Wt.%) ratio for of the serpentinite rock. (**b**) variation of SiO_2_vs. FeO^*^+MgO for the serpentinite rocks after (Coleman, 1977^50^). (**c**) AFM ternary diagram for serpentinite rocks after (Coleman, 1977^50^). (**d**) MgO-CaO-Al_2_O_3_ ternary diagram for serpentinite rocks after (Coleman, 1977^50^). (**E**) Al2O3 vs. CaO distribution fields for the studied serpentinites after (Aumento and Laubat, 1971^51^).

The RO`/SiO2^43^ shows a wide ranges of variation (1.37–1.64%) and averaged 1.59%. This ratio reflects the variation in the pyroxene and olivine contents in the original parent ultramafic body, which as mainly peridotite. The binary diagram of the MgO/SiO2 ratio with the olivine content (Fig. 6a) shows that, olivine content increases with the increasing of MgO/SiO_2_ ratio. The average of MgO/SiO2 ratio of serpentinized dunite^44^ is 1.234 while in serpentinized harzburgite is 1.06 and for theoretical serpentinite is 1.01. The average MgO/SiO_2_ of the analyzed samples ranges from 0.872 to 1.014 with an average of 0.89 for the more pyroxene-rich harzburgite and 0.98 for the olivine-rich harzburgite. It is appeared that the present serpentinite analyses plot within the ultramafic cumulates and average metamorphic peridotite (Fig. 6b, c and d). As whole, the studied serpentinite fall within the low temperature alpine type field (Fig. 6E). Gulacar and Delalaye^45^ used the Ni/Co ratio of serpentinite analyses to characterize the dunite and peridotite of Bushveld complex (8.07%) in one side and those of Alpine type (20.97%) on another side. Accordingly, the studied serpentinite (Ni/Co is 51%) possible belongs to the Alpine type.

Loss on ignition (LOI) is employed by Malakhov^46^ to determine the degree of serpentinization where, 2.13, 6.05 and 11.67% are recorded for slightly, moderately and intensively serpentinized ultramafic rocks. The obtained LOI values for the studied serpentinite (11.70–19.20 and Av. of 14.31%) suggest intensive serpentinization. All the samples of serpentinite and talc-carbonate exhibit elevated Cr and Ni contents. Cr_2_O_3_ ranges from 0.23 to 0.41% and Ni from 0.28 to 0.33%. Ni occurs mainly in sulphide (pentalandite) and in the silicates and carbonates in substitution for iron and magnesium. Cr_2_O_3_ occurs chiefly in chromite, ferritchromite and Cr-magnetite. The Ni and Cr contents of the ultramafic rocks are normally in the range of 0.3 and 0.4% respectively^47–49^. The Ni and Cr contents of the analyzed samples are generally the same like this, suggesting that Cr and Ni were released and incorporated in serpentinite rock during the serpentinization.

Co, Zn and Sr occur in very small amount. Co is contained probably in silicate minerals in substitution for Mg. Zn is contained in the chromite structure. Sr shows a marked mobility and fluctuation in the serpentinite rock samples and tends to be enriched in Ca-carbonate bearing serpentinite.

**Table 3:** Chemical analyses (major in Wt% and trace elements in ppm), norm calculation and geochemical parameters of the analyzed serpentinite and talc-carbonate rocks:

Rock type	Serpentinite rocks											Talc-carbonate			
Oxides	22	21	20	3	37	38	1	4	6	19	Av.	8	7	5	Av.
SiO_2_	43.37	42.75	41.41	39.33	38.89	38.63	37.89	35.89	35.25	32.89	38.63	34.27	21.67	32.79	29.58
A1_2_O_3_	0.64	0.53	1.17	0.90	0.57	0.54	0.86	0.65	0.72	0.39	0.70	0.83	0.62	0.50	0.65
Fe_2_O_3_	1.70	2.18	3.00	2.21	4.07	4.51	4.51	2.66	2.51	2.50	2.95	1.91	2.46	4.17	2.85
FeO	2.45	1.95	2.92	2.76	1.95	1.74	1.94	3.33	3.24	2.16	2.44	3.38	4.81	0.51	2.90
TiO_2_	0.04	0.03	0.02	0.03	0.02	0.02	0.03	0.03	0.02	0.03	0.03	0.02	0.03	0.03	0.03
CaO	0.03	0.17	0.58	0.59	0.88	0.66	2.51	2.68	5.19	7.16	2.05	0.14	035	12.97	4.49
MgO	37.82	38.32	37.78	38.07	37.98	37.92	36.59	36.40	34.00	32.76	36.76	34.31	34.99	23.62	30.97
Na_2_O	0.04	0.06	0.04	0.05	0.02	0.04	0.09	0.05	0.02	0.02	0.04	0.05	0.05	0.16	0.09
MnO	0.11	0.06	0.12	0.08	0.09	0.10	0.09	0.13	0.14	0.22	0.11	0.05	0.17	0.26	0.16
S	< 0.01	0.01	< 0.01	0.01	0.01	0.01	0.06	0.01	< 0.01	< 0.01	0.01	0.01	0.02	0.05	0.03
Cr_2_O_3_	0.34	0.32	0.35	0.33	0.38	0.41	0.38	0.34	0.35	0.23	0.43	0.33	0.49	0.24	0.35
Ni	0.30	0.33	0.33	0.30	0.33	0.31	0.31	0.28	0.29	0.32	0.31	0.32	0.35	0.26	0.31
LOI	11.70	12.00	11.80	13.80	13.50	13.20	14.50	16.10	17.30	19.20	14.31	23.80	32.40	23.20	26.47
Sum	98.54	98.72	99.52	98.46	98.69	98.09	99.40	98.55	99.03	97.88	98.77	99.42	98.41	98.76	98.88
As	< 5	< 5	< 5	< 5	< 5	< 5	< 5	< 5	< 5	< 5	< 5	10	98	< 5	8
Pb	< 5	< 5	< 5	< 5	< 5	< 5	< 5	< 5	< 5	< 5	< 5	< 5	< 5	< 5	< 5
Zn	32	27	36	27	47	39	30	30	35	35	34	24	53	36	38
Co	46	55	67	48	67	70	64	62	69	60	61	64	98	59	74
V	3	< 1	17	11	8	6	4	10	11	4	7	3	45	20	23
La	< 5	< 5	< 5	< 5	< 5	< 5	< 5	< 5	< 5	< 5	< 5	< 5	< 5	< 5	< 5
Nd	< 5	< 5	< 5	< 5	< 5	< 5	< 5	< 5	< 5	< 5	< 5	< 5	< 5	< 5	< 5
Ce	8	< 5	< 5	< 5	< 5	< 5	6	< 5	< 5	< 5	1	7	< 5	< 5	2
Ga	< i	< i	< 1	< 1	< 1	1	< 1	< 1	< 1	< 1	< 1	< 1	1	< 1	< 1
So	< i	i	i	2	1	< 1	2	< i	5	3	2	< 1	< i	3	1
Nb	i	< i	< i	1	i	< i	1	2	< 1	2	2	< 1	< i	2	1
Zr	< i	< i	< 1	< 1	< i	< i	< 1	< 1	< 1	2	< 1	< 1	< 1	< i	< 1
Y	1	< i	< i	< 1	< i	i	< 1	< 1	< 1	< 1	< 1	< 1	3	i	1
Sr	i	3	4	4	14	18	23	19	18	53	16	1	5	59	22
U	< 5	< 5	< 5	< 5	< 5	< 5	< 5	< 5	< 5	< 5	< 5	< 5	< 5	< 5	< 5
Rb	< 1	< 1	< 1	< 1	< 1	< 1	< 1	< 1	1	1	< 1	< 1	< 1	< 1	< 1
Th	< 1	< 1	< 1	1	< 1	< 1	3	1	2	< 1	< 1	1	< 1	< 1	< 1

Parent rock	Pyroxene-rich harzburgite			Olivine-rich harzburgite.											
Fo	40.61	43.82	47.35	55.31	55.82	56.36	54.33	62.24	55.55	59.10	53.05				
Fa	2.15	1.83	3.00	3.27	2.35	2.12	2.35	4.65	4.34	3.19	2.93				
Olivine	*r* ~ 42.76	45.65	50.35	58.58	58.17	58.48	56.68	66.89	59.89	62.29	55.97				
En	54.62	52.38	46.97	39.97	40.30	40.16	41.70	31.08	2.65	1.66	35.08				
Fs	2.62	1.97	2.69	2.12	1.53	136	1.62	2.10	37.46	24.31	7.78				
Pyroxene	57.24	54.35	49.65	41.42	41.83	41.52	43.32	33.18	40.11	37.71	44.03				
*MgO/SiO_2_	0.87	0.90	0.91	0.97	0.98	0.98	0.97	1.01	0.97	1.00	0.96				
*Fe_2_O_3_/FeO+Fe_2_O_3_	40.96	52.78	50.68	44.44	67.56	72.16	68.14	44.40	43.60	53.60	53.83	36.11	33.84	89.1	49.57
*CO_2_	0.05	0.27	0.91	1.11	1.65	1.24	4.70	5.02	8.14	11.23	3.43	–	–	–	–
*H_2_O	11.92	12.05	11.21	13	12.07	12.15	10.02	11.45	9.52	8.21	11.16	–	–	–	–
*«RO7SiO_2_	1.37	1.41	1.46	1.54	1.57	1.59	1.56	1.64	1.56	1.60	1.53	–	–	–	–
**H_2_O/SiO_2_	0.92	0.94	0.90	1.1	1.04	1.05	0.88	1.06	0.90	0.83	0.96	–	–	–	–
“MgO/MgO+FeO + MnO	0.94	0.394	0.92	0.93	0.92	0.92	0.92	0.92	0.91	0.92	0.942	–	–	–	–

*Wt% ratios.

**Molar ratios.

### Rare earth elements (REE)

Four samples analyzed for REE by Inductively Coupled Plasma Spectrometer (ICP-MS). The samples are plotted on a chondrite normalized diagram (Fig. 8a) using the chondrite values of Boynton^52^. Generally, the analyses are depleted in REE relative to the chondrite REE abundances (Table 4). Hathout^53^ studied the REE abundances of ultramafic rocks of both G. El- Mueilih and G. Handarba and reported that the rocks which might have dunitic character contain lower REE contents than those have peridotitic tendency.

The pyroxene-rich harzburgite samples (22, 20) have REE abundances controlled by the modal amount and REE content of olivine, orthopyroxene and plagioclase in the original ultramafic body. The REE of the olivine-rich harzburgite samples (19, 37) are dominated by the modal amount of olivine relative to orthopyroxene. Since these minerals have depleted light REE, the patterns (Fig. 8a) show same character and further show much depletion in heavy REE due to the talc-carbonate alteration. The carbonate bearing samples are slightly depleted in light REE and strongly in the heavy REE. This reflects that the REE are affected (mobilized) by addition of CO_2_ and leaching of Mg and Ca from the parent rocks to form carbonates.

**Table 4:** Rare Earth elements concentrations of serpentinite rocks together with the normalized values:

Analysis	Serpentine rocks				Chondrite values Boynton, 1984	Normalized values			
Rock type	Pyroxene-rich Harzburgite		Olivine-rich Harzburgite			Pyroxene-rich harzburgite		Olivine-rich harzburgite	
Samples No.	22	20	37	19		22	20	37	19
La	0.119	0.166	0.131	0.178	0.31	0.384	0.535	0.423	0.574
Ce	0.262	0.0456	0.22	0.259	0.808	0.324	0.564	0.272	0.321
Pr	0.033	0.079	0.019	0.023	0.122	0.270	0.648	0.156	0.189
Nd	0.168	0.402	0.069	0.091	0.6	0.280	0.670	0.115	0.152
Sm	0.047	0.152	< 0.019	< 0.019	0.195	0.241	0.779	–	–
Eu	0.017	0.011	< 0.002	0.002	0.0735	0.231	0.150	–	–
Gd	0.075	0.175	< 0.019	< 0.019	0.259	0.290	0.676	–	–
Tb	0.011	0.031	< 0.002	< 0.002	0.0474	0.232	0.654	–	–
Dy	0.028	0.213	0.008	0.008	0.322	0.255	0.661	0.025	0.025
Ho	0.017	0.047	< 0.004	< 0.004	0.0718	0.237	0.655	–	–
Er	0.039	0.139	< 0.004	< 0.004	0.21	0.186	0.662	–	–
Yb	0.034	0.174	< 0.011	< 0.011	0.209	0.163	0.833	–	–
Lu	< 0.008	0.032	< 0.008	< 0.008	0.0322	–	0.994	–	–
Sum	0.904	2.077	0.447	0.561	3.2599	3.092	8.481	0.990	1.287
					La/Yb	2.36	0.64	–	–

### Mode of occurrence of talc deposit

The talc deposit is poorly exposed on the surface. Good exposures are confined to the shear zones. The talc deposit varies greatly in shape and attitude occurring in the form of sporadic pod-shaped or lenticular, sheet-like masses, which pinch and swell within a host of serpentinite. Talc bodies are also variable in extent. The average of these veins reaches 20–30 m with general trend in N20°W and their width ranges between 2 and 4 m. They are steeply dipping 66°NW or sometimes vertical. Talc samples have grey, light green and dark green colors. Talc usually has massive nature, but sometimes changes to friable and shows schistose appearance along the contact with the host rocks. At the outer part of massive talc rock, a thin zone of chlorite rock (which is reported as black wall in literature^54^) is encountered. This chlorite shell separates the talc body from all country rocks except for talc-carbonate.

### Mineralogy of talc mineralization

#### Results of XRD

Nine representative samples from different talc ore and talcified rocks were analyzed by Cu K radiation (= 1.5406) on randomly oriented powdered specimen. The samples were scanned from 2–70° and presented in Table 5.

Identification of minerals was made by comparing the d-spacing and intensities of minerals with those reported in the JCPDS powder diffraction file, computer programme (Diffrac-At) and data in literatures. Identification of minerals were judged to be present in major, minor and rare by using the intensity of the main reflection from each mineral (Table 5). Accordingly, three ore types are identified. They are talc, tremolite-talc and chlorite-talc ore types. Although, the talc samples occur in a fairly pure form, a few percent of chlorite, tremolite, chrysotile, antigorite and chromite are encountered.

**Table 5:** Mineralogical composition of the Talc deposit as obtained by XRD investigations:

Ore type	No.	Major	Minor	Rare
1-pure talc	10	Talc	–	Chlorite
	9	Talc	–	Chlorite
	30	Talc	–	Chlorite, Antigorite
	11	Talc	–	Chlorite, Chromite
	25	Talc	–	Chlorite, Lizardite, Chromite
	15	Talc	–	Chlorite
	28	Talc	–	Chlorite
2-Tremolite-talc	18	Talc	Tremolite	Chlorite
3- Chlorite-talc	27	Chlorite, talc	Tremolite	

#### Microscopic examinations of Talc deposit

The petrographic investigations of the talc ore revealed that the main mineral assemblage found is talc, chlorite and tremolite in decreasing order of abundance. Chromite, chrysotile, lizardite are rarely detected. On the basis of predominant minerals of the studied samples, three ore types have been identified namely, pure talc, tremolite-talc and chlorite-talc.

1. *Pure talc ore type*: It shows a greater abundance than the other ore types. It is fine grained, massive and has pale to dark greyish green color. The rock consists mainly of talc (90% or more) with minor chlorite and tremolite(less than 10%). Relict minerals of parent rocks (chromite, magnetite and lizardite) occur as accessories.
2. *Tremolite-talc ore type*: This is an uncommon ore type. It is a fine grained, sometimes has a fibrous nature and schistose appearance. It has pale yellowish green color with patches of dark green chlorite. It consists essentially of talc (80% or more) together with few proportions of tremolite and chlorite. Chromite is common accessory.
3. *Chlorite-talc ore type*: It is grained rock, massive and has patchy coloration from pale brown, greyish green and dark green colors. The mineral assemblage includes chlorite and talc (nearly in equal proportions). Tremolite and chrysotile are also detected.

**Fig. 7:**
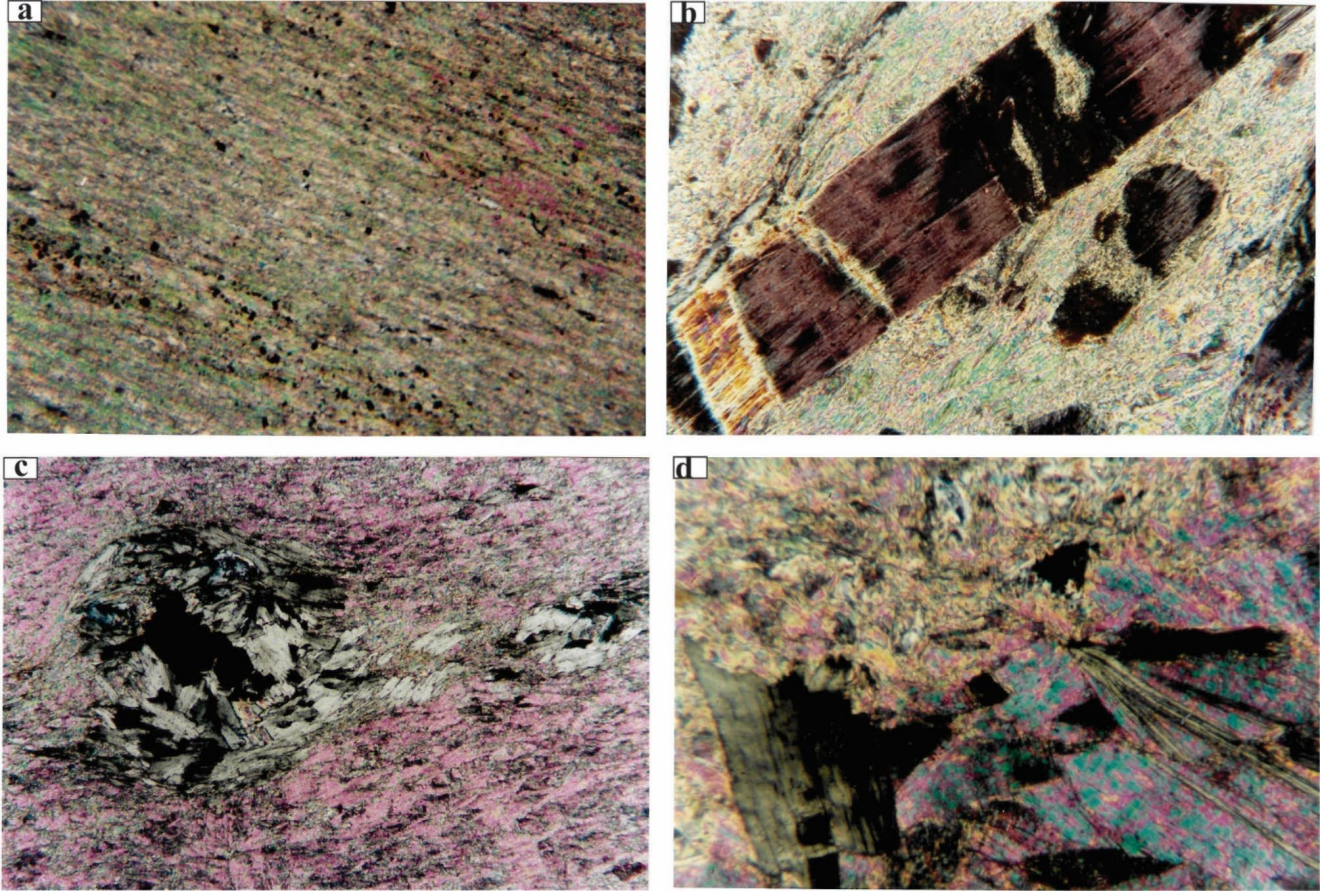
(**a**) photo showing preferred orientation of fine talc shreds and disseminated fine opaques (the field of view 4 mm across, XPL), (**b**) A characteristic alteration model tremolite→chlorite→talc. Chlorite exhibits the columnar form of tremolite and in turn is altered to talc (the field of view 1 mm across, XPL), (**c**) A typical alteration scheme of iron-bearing chlorite to talc. Iron hydroxides possibly are released chlorite is presumably formed from the released Al during the alteration of chromite (the field of view 2 mm across, XPL), (**d**) alteration of chlorite to talc with remnants from cleavage planes and crystal from in matrix of talc (the field of view 1 mm across, XPL).

The brief petrographic character of each identified minerals will be summarized in the following:

*Talc (7a-d)* always occurs as fine shreds, dense fibers and is rarely observed as plates Fig. 7a. Perfect cleavage, straight extinction and high interference color are characteristic features of talc. Talc is derived from different minerals amongst which, are serpentine, chlorite and tremolite. The conversion of serpentine to talc is clearly observed and relics from fresh serpentine remain in the talc rock matrix. Talc sometimes has flaky and fan-shaped crystals which pseudomorph chlorite. The alteration of chlorite to talc is accompanied by introduction of iron oxide Fig. 7d. It is also observed actively replacing and corroding tremolite boundaries and cores.

*Chlorite* occurs in the form of fine anhedral plates and sometimes is found as tabular and fan-shaped crystals in the form of discrete grains and/or aggregates. It has a pale green color, weak pleochroism and anomalous greenish brown and greyish blue interference color. It is commonly formed during the hydrothermal alteration of the mafic minerals in the parent ultramafic rocks. It is clearly seen replacing tremolite partly and completely and shows the typical elongated and prismatic crystal form giving rise a typical example of tremolite →chlorite → talc transformation Fig. 7b.

*Tremolite* is an uncommon mineral and occurs in the form of fine to medium grained prismatic and elongated crystals. Tremolite is found as a result of metamorphic alteration of primary pyroxene minerals present in the ultramafic rocks found in the studied area. In turn, it is readily seen replaced by and altered to both chlorite and talc. Actinolite is rarely observed in few samples. It has light green color and occurs in the form of elongated crystals.

*Lizardite* and chrysotile are very rare minerals observed in the talc samples. They occur as relics after complete talcification of serpentinite rocks. Chrysotile occurs in the form of small veinlets cutting across a matrix of talc and chlorite in the chlorite-talc ore type.

#### Opaque minerals of talc deposit

The opaque mineralogical study reveals the presence of chromite, chromium magnetite, magnetite and goethite in the talc deposit samples. These opaque minerals were in the parent serpentinized ultramafic rocks or from the materials released from the alteration of the chlorite to talc.

*Chromite* is relatively abundant in the talc deposit and occurred as primary mineral derived from the parent ultramafic rock. It occurs in the form of massive, fine to medium grained (0.01–2.3 mm) and has a mostly oval to elongate crystal form. It has dark grey to brownish grey color, usually with blood red tint. As a result of tectonic events accompanied to the emplacement of serpentinized ultramafic rocks, most grains show marked cataclastic hair- like cracks Fig. 5e. The cracks are partially cemented by talcified materials Fig. 7c. The hardness of chromite as measured in term of Vickers Hardness Number (VHN) is 1139.

*Magnetite* is characterized by two modes of occurrences. The first is pure magnetite which occurs in the form of small discrete tabular grains and as stringers and trains of grains throughout the talc matrix. It has euhedral to anhedral polycrystalline aggregates. It has grey color, commonly with brownish tint. The second is magnetite rich in chromium (assigned chromium magnetite). It often shows some degree of alteration and contains lamellae from martite arranged in triangular patterns. Two possible origins of magnetite are considered here, magmatic and secondary. The latter is probably formed from the iron released during the talcification of iron-rich minerals as chlorite.

*Goethite* occurs as a secondary mineral. It is originated by more or less hydration of magnetite. The alteration of magnetite to goethite reaches up to later stages of the replacement. Generally, it has dull grey color. It shows a distinct polarization color (blue grey) and internal reflection (brownish yellow to reddish brown).

#### Mineral chemistry of Talc deposit

A detailed study on the mineral chemistry of the talc deposit was done using polished thin sections (for transparent minerals). More than ten samples were selected for the analysis by Microprobe Electron Microscope. The obtained analyses are listed in Table 6. The iron is given as FeO* and it was recalculated to F e^2+^ and F e^3+^ using the computer program (Mica) for structural formula calculations. The compositional variations of talc, tremolite, chlorite and chrysotile in the specimens from the different ore types are presented in the following;

*Talc* is chemically homogeneous with occasional little chemical variation. Most of the minor elements of talc were inherited from the parent rock while others were introduced to the system from an external source. Chromium, nickel and iron are known to be enriched in ultramafic rocks unlike volcanic ones. The structural formula the basis of 14 oxygen is Mg_2.931_ Fe^2 +^ _0.054_ Fe ^3+^_0.079_Ni _0.012_ Mn _0.001_ Cr _0.001_ Si _3.93_ O _10_ (OH) _2_. Therefore, the content of iron and nickel in the talc under study (Table 6) are much higher than that derived from carbonatized metavolcanics (Marahique area) with a formula, Mg_3.02_ Cr _0.001_ Si _3.97_ O _10_ (OH)_2_^57^.

**Fig. 8:**
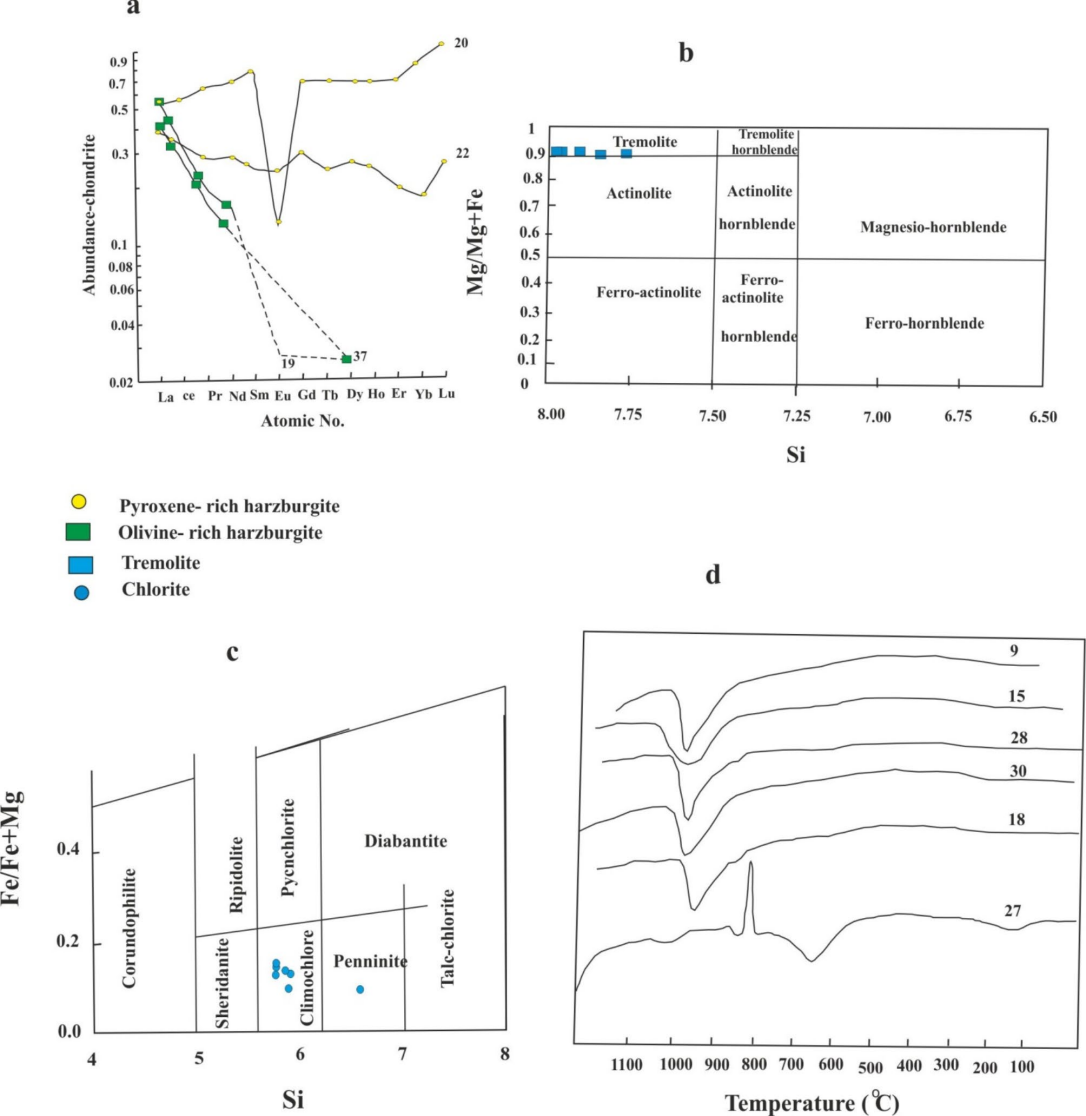
(**a**) Chondrite- normalized (Boynton, 1984^52^) rare earth elements diagram of the serpentinite. (**b**) Nomenclature of detected amphibole mineral according to Leake (1978^55^). (**c**) Fe/Fe + Mg –Si plot of chlorite analyses (After Hey, 1959^56^). (**d**) the thermal behaviors of the talc samples (under heating program from room temperature to about 1150^c^). Samples no 9, 15, 28 and 30 belong to talc ore, no 18 is tremolite-talc ore and no. 27 is chlorite-talc ore.

*Tremolite* is characterized by the elevated iron content, where the FeO* content ranges from 4.44 to 5.5% which is more than that present in its structure^58^. This is due to the same reason for the high elevated iron in most of the detected silicate minerals which is the parent ultramafic rocks. The average composition of tremolite is 57.42% SiO_2_, 0.72% Al_2_O_3_ 4.87% FeO*, 12.28% CaO and 22.19% MgO. Leake^55^ classified the clino-amphiboles on the basis of Mg / Mg + Fe ratio and Si content (of which Ca + Na are more than 1.34 and Na less than 0.67). Accordingly, both tremolite and actinolite are represented (Fig. 8b). Tremolite has 7.952 Si, 0.906 Mg/Mg + Fe and a structural formula of Ca_1.816_ (Mg_4.611_ Fe^2 +^ _0.477_Fe^3+^_0.050_Mn_0.040_ Cr_0_._012_) Si_7.952_ Al_0.034_ O_22_(OH, F)_2_. Actinolite has 7.814 Si, 0.984 Mg/Mg + Fe and a chemical formula of Ca 1.798 (Mg _4.447_ Fe ^2+^ Fe ^3+^_0.529 0.082_ Mn _0.04_ Cr _0.050_) Si_7.814_ Al_10.242_ O_22_ (OH, F)_2_. The calculated formula clearly reflects that the replacement of Mg by Fe^2+^ exceeds the Si by Al.

*Chlorite* is compositionally homogeneous and shows a narrow range of chemical variation in the contents of SiO_2_, MgO, FeO* and Al_2_ O_3_. The main major substitutions in the chlorite are Si by Al and Mg by Fe. The studied chlorite has the following structural formula, (Mg _8.70_ Fe ^2+^_1.19_ Al^vi^_1.846_) (Si _5.987_ Al _2.013_) O_20_ (OH)_16_.

The A1^iv^ values of chlorite has a great significant in determining the temperature at which chlorite is formed^59,60^. The equation for calculating temperature is T^o^C = 106 Al^iv^_1.846_ Using this equation, the temperature of chlorite formation ranges from 164 to 251 with an average of 231 °C.

The A1^IV^ Al^VI^ ratio in the analyzed chlorite ranges from 0.97 to 1.38 and averaged 1.11, as mentioned by Zang and Fyfe^61^ wherever the ratio is close to unity the charge-balance of Al ^VI /^Si replacement is accomplished by Al ^VI^ replacing Fe and/ or Mg in the octahedral sites. Also, suggesting the lower Fe ^3+^ in chlorite. The content of Si and Fe/Fe + Mg ratio has been used by Hey^56^ in classification and nomenclature of chlorite. Chlorite lies mainly in the zone of clinochlore and only one sample in the pennine field (Fig. 8c). In general, it is Mg-rich type reflecting the fact that it is linked to the bulk rock composition and affected by the Mg-metasomatic process.

*Chrysotile* has the following formula Mg _5.272_ Fe ^2+^_0.425_ Cr _0.007_ Si _4.254_ Al _0.017_ O_10_ OH _8_. The (Fe ^2+^ Mg^2+^ /Fe ^3+^ Al^3+^) ranges from 144 to 203% with an average of 174% which is nearly similar to that reported by Page^62^ for chrysotile (113%). Also, the chemical characters of studied mineral (low Al_2_ O_3_ and high H_2_O contents) are similar to that reported for chrysotile by Moody^63^.

**Table 6:** The mineral chemistry and structural formula of Talc and associated tremolite, actinolite, chlorite and Chrysotile.

Minerals	Talc									Chlorite							Actinolite		Tremolite			Chrysotile			
No.	WS6	WS17	WS21	CC4	CC7	CC10	G17	ZX5	ZX12	CC12	ZX3	ZX9	ZX10	WS8	WS12	WS20	WS5	WSU	WS7	ZX8	ZX11	CC19	CC11	CC15	CC18
SiO_2_	62.12	59.14	61.49	62.86	63.19	62.62	63.50	63.37	61.86	34.22	31.06	30.49	31.01	30.56	31.24	31.05	55.96	57.27	57.34	58.01	58.51	42.75	43.86	45.82	42.64
AI_2_O_3_	0.00	0.00	0.00	0.00	0.00	0.00	0.00	0.00	0.00	11.51	18.87	17.88	18.27	17.92	19.08	16.83	1.87	1.09	0.55	0.00	0.07	0.00	0.00	0.33	0.26
TiO_2_	n.d	n.d	n.d.	n.d.	n.d.	n.d.	n.d.	0.07	0.00	n.d.	0.12	0.00	0.04	n.d.	n.d.	n.d.	n.d.	n.d.	n.d.	0.00	0.00	0.00	n.d.	n.d.	0.00
FeO	2.56	**2.66**	**2.45**	2.72	**3.34**	2.28	1.25	2.84	2.91	5.85	8.59	8.89	8.03	8.14	8.03	6.53	5.50	5.08	4.67	4.44	4.66	3.06	8.37	4.36	4.94
CaO	0.00	0.08	0.06	0.00	0.05	0.00	n.d.	0.00	0.00	0.09	0.00	0.07	0.02	0.05	0.02	0.06	12.15	12.17	12.35	12.36	12.35	0.04	0.25	0.14	0.10
MgO	30.22	33.33	31.55	30.56	31.06	30.86	32.24	30.62	29.85	33.11	29.77	30.01	30.26	29.33	30.23	30.41	21.22	22.10	22.24	22.79	22.61	39.73	31.42	35.55	39.28
MnO	0.06	0.02	0.00	0.02	0.05	0.09	n.d.	0.00	0.00	0.10	0.16	0.09	0.18	0.10	0.10	0.04	0.31	0.00	0.44	0.23	0.34	0.00	0.01	0.00	0.10
Cr_2_O_3_	0.00	0.02	0.00	0.04	0.00	0.01	n.d.	0.00	0.00	1.16	1.36	1.21	0.63	1.12	0.20	2.15	0.35	0.56	0.15	0.06	0.10	0.08	0.11	0.12	0.06
ZnO	n.d.	0.03	0.00	n.d.	n.d.	n.d.	n.d.	0.00	0.03	0.11	0.12	0.00	0.02	n.d.	0.00	0.10	n.d.	0.00	n.d.	0.00	0.00	0.00	n.d.	0.07	n.d.
NiO	n.d.	n.d.	n.d.	n.d.	n.d.	n.d.	n.d.	0.20	0.24	n.d.	0.24	0.27	0.14	n.d.	n.d.	n.d.	n.d.	n.d.	n.d.	0.00	0.22	0.17	n.d.	n.d.	0.18
Sum	94.96	95.28	**95.55**	96.21	97.69	**95.86**	96.99	97.10	94.89	86.16	90.28	88.91	88.58	87.21	88.89	87.17	97.37	98.26	97.74	97.89	98.86	85.84	84.02	86.39	87.56
Structural formula																									
Si	3.976	3.735	3.891	3.974	3.936	3.963	3.948	3.975	3.973	6.631	5.836	5.804	5.897	5.921	5.909	5.912	7.775	7.852	7.908	7.970	7.979	4.081	4.473	4.442	4.022
Al^IV^	0.00	0.00	0.00	0.00	0.00	0.00	0.00	0.00	0.00	1.369	2.164	2.196	2.103	2.079	2.091	2.088	0.307	0.177	0.089	0.00	0.013	0.00	0.00	0.038	0.029
Sum			–	–			*	–	–	8.000	8.000	8.000	8.000	8.000	8.000	8.000	–	–	–	–	–	–	–	–	–
Al^VI^		–		–	–	–	–		–	1.260	2.016	1.816	1.990	2.013	2.144	1.688	–	–	–	–	–	–	–	–	–
Ca	0.00	0.005	0.004	0.00	0.003	0.00	–	0.00	0.00	0.019	0.00	0.014	0.00	0.011	0.00	0.012	1.809	1.787	1.825	1.819	1.805	0.004	0.027	0.015	0.010
Mg	2.883	3.137	2.976	2.879	2.884	2.911	2.987	2.862	2.857	9.562	8.338	8.516	8.576	8.470	8.523	8.915	4.395	4.498	4.571	4.667	4.596	5.652	4.776	5.138	5.523
Fe^2+^	0.090	0.00	0.00	0.093	0.046	0.047	0.00	0.105	0.102	0.948	1.349	1.218	1.269	1.319	1.270	0.965	0.535	0.523	0.461	0.457	0.513	0.244	0.714	0.353	0.390
Fe^3+^	0.047	0.120	0.129	0.051	0.128	0.073	0.065	0.044	0.054	0.00	0.00	0.198	0.008	0.00	0.00	0.075	0.105	0.059	0.077	0.053	0.019	0.00	0.00	0.00	0.00
Ni	–	–	–	*	*		–	0.100	0.012	–	0.036	0.041	0.021	–	–	–	–	–	–	–	0.025	0.013	–	–	0.001
Ti	–		**“**	**-**			–	0.004	0.00	–	0.017	0.00	0.00	–	–	–		–	–	–	0.00	0.00	–	–	0.00
Mn	0.003	0.00	0.00	0.001	0.003	0.005	–	0.00	0.00	0.017	0.026	0.014	0.029	0.016	0.015	0.006	0.036	0.044	0.052	0.027	0.040	0.00	0.001	0.00	0.008
Cr	0.00	0.001	0.00	0.002	0.00	0.001		0.00	0.00	0.178	0.201	0.182	0.095	0.172	0.029	0.324	0.039	0.060	0.017	0.007	0.011	0.006	0.009	0.009	0.004
Zn	**”**	–	0.00	–	**“**	**“**	**“**	0.00	0.00	0.016	0.017	0.00	0.00	**“**	0.00	0.014	–	0.00		–	0.00	0.00	–	0.005	–

### Results of thermal analyses

In order to study the thermal behavior of the mineral phases, 6 samples were selected for the thermal analysis. The DTA curves were made simultaneously using a Stanton-Redcroft DTA instrument. The samples were heated from room temperature to 1150 ^o^ C Thermal analyses curves for all the analyzed samples are given in (Fig. 8d). On the basis of thermal curves, the following minerals were detected and arranged in decreasing order of abundance, talc, chlorite, tremolite, chrysotile, dolomite and magnesite. A brief description of the thermal behavior of the principal minerals is given in the following.

*Talc*, the DTA curves of talc show a single, strong and very endothermic peak situated between 900 and 1000 ^o^C. This endothermic is attributed to the removal of hydroxyl bonded to magnesium. This result is similar to that reported by Mackenzie^64^ and Evans and Guggenheim^65^.

*Chlorite*, the DTA curves of chlorites generally consists of two distinct endothermic peaks. The removal of interlayer hydroxyl sheet is generally associated with a broad endothermic peak at moderate temperature (645^o^C). The removal of silicate hydroxyl sheet is associated with a high temperature endothermic peak (835^o^C). On further heating, at temperature about 860^o^C, a moderate exothermic recrystallization peak appears on the thermal curves. The studied chlorite shows similar thermal behavior for trioctahedral chlorites^66^ where they recorded 600–650^o^C for endothermic peak and 800–900 °C for endothermic-exothermic peak system.

*Tremolite*, the DTA curves of tremolite show a weak and broad endothermic peak situated at 1000–1045^o^C Thermal effect specific to tremolite is given by removing the hydroxyl groups from its structure.

*Chrysotile*, on heating two thermal effects of chrysotile is recorded. The first is endothermic (dehydroxylation) at 650–750^o^C. The second is exothermic (structural reorganization) at 800–830^o^C.

### Chemical characteristics of talc deposit

Nine samples from different ore types were selected for whole rock geochemistry. The results of chemical analyses are presented in Table 7. The chemical data of talc deposit reveals that the summation of talc components (SiO_2_ + MgO + H_2_O) is 92.685, while that of impurity oxides (Al_2_O_3_ + CaO + Fe_2_O_3+_ FeO) is 5.56%.

Based on the Al_2_O_3_ content, Romanovuch^67^ classified the talc deposits into two groups: a) aluminous talc Al_2_O_3_ > 4%) and b)- non- aluminous talc (Al_2_O_3_ < 4%). In the studied deposits, it is noticed that samples from pure talc and tremolite talc lie within the non-aluminous talc group whereas the chlorite talc type lie within the aluminous talc group. Romanovuch^68^ classified the talc deposits according to the total iron content into: ferruginous talc (Fe_2_O_3_ > 2.75%) and non-ferruginous talc (Fe_2_O_3_ < 2.75%) Accordingly, the pure talc and tremolite talc types are located within the category of non-ferruginous talc, while the chlorite talc types are located within the ferruginous talc group.

The talc deposits show higher concentrations of Cr_2_ O_3_ (av. 0.24%), Ni (av. 0.23%) and Co (av. 42ppm). Generally, Cr_2_O_3,_ Ni and Co show very insignificance decrease from serpentinite rocks (av. 0.34% Cr_2_ O_3_ 0.3% Ni and 61 ppm Co) and talc carbonate (av. 0.35% Cr203, 0.31 Ni and 74 ppm Co) compared with talc deposit samples. No appreciable movement of Cr, Ni and Co have occurred during talcification process and appear to be more closely related to their contents in the parent ultrabasic rocks.

**Table 7:** Major (wt%) and trace (ppm) elements contents of the different Talc ore. For comparison, the average contents of the parent rocks are included.

Rock Type	Talc ore deposit									Talc-carbonate	Serpentinite rock
	Talc							Tremolite Talc	Chlorite talc		
Oxide	10	9	30	11	25	15	28	18	27	Av.	Av.
SiO_2_	61.48	61.25	60.46	60.36	60.33	59.82	59.01	58.79	44.17	29.58	38.63
A1_2_O_3_	0.11	0.29	0.25	1.09	0.14	0.14	1.29	1.11	5.04	0.65	0.70
Fe_2_O_3_	0.48	0.41	0.68	0.21	0.81	2.43	0.27	0.93	2.91	2.85	2.95
FeO	2.71	3.19	2.62	3.02	2.93	2.16	3.24	3.50	3.44	2.90	2.44
TiO_3_	0.00	0.00	0.00	0.07	0.00	0.00	0.03	0.00	0.04	0.03	0.03
CaO	0.09	0.02	0.02	0.03	0.10	0.04	0.05	1.31	3.68	4.49	2.05
K_2_O	0.00	0.00	0.00	0.00	0.00	0.00	0.00	0.00	0.02	0.00	0.00
MgO	29.42	28.90	28.78	28.78	29.05	29.32	28.65	27.79	28.60	30.97	36.76
Na_2_O	0.04	0.06	0.08	0.07	0.07	0.09	0.10	0.10	0.20	0.09	0.04
MnO	0.02	0.03	0.03	0.07	0.03	0.02	0.05	0.07	0.17	0.16	0.11
S	< 0.01	,0.01	< 0.01	< 0.01	< 0.01	0.01	< 0.01	0.01	0.02	0.03	0.01
P_2_O_5_	0.00	0.00	0.00	0.00	0.00	0.00	0.00	0.00	0.00	0.00	0.00
Cr_2_O_3_	0.25	0.26	0.21	0.31	0.20	0.24	0.26	0.22	0.24	0.35	0.34
Ni	0.24	0.25	0.24	0.24	0.22	0.20	0.23	0.22	0.24	0.31	0.31
LOI	4.77	4.64	4.86	4.68	5.11	4.80	4.90	5.18	10.20	26.47	14.31
Sum	99.61	99.30	98.23	98.93	98.99	99.27	98.08	99.23	98.07	98.88	98.68
As	< 5	< 5	< 5	< 5	< 5	< 5	< 5	< 5	< 5	36	< 5
Pb	< 5	< 5	< 5	< 5	< 5	< 5	< 5	< 5	< 5	< 5	< 5
Zn	34	42	42	51	33	27	64	76	56	38	34
Co	42	48	37	46	41	32	43	47	51	74	61
V	13	11	4	22	4	11	5	4	16	23	7
La	< 5	< 5	< 5	< 5	< 5	< 5	< 5	< 5	< 5	< 5	< 5
Nd	5	< 5	16	< 5	< 5	< 5	< 5	< 5	< 5	< 5	< 5
Ce	< 5	< 5	15	< 5	< 5	< 5	< 5	< 5	< 5	2	1
Ga	1	1	< 1	1	< 1	1	3	< 1	13	< 1	< 1
Sc	< 1	< 1	< 1	1	< 1	< 1	< 1	< 1	2	1	2
Nb	2	< 1	< 1	1	< 1	1	< 1	< 1	< 1	1	2
Zr	< 1	< 1	< 1	< 1	< 1	1	< 1	< 1	< 1	< 1	< 1
Y	< 1	1	< 1	< 1	< 1	< 1	< 1	< 1	2	1	< 1
Sr	5	1	1	< 1	1	2	< 1	6	9	22	16
U	< 5	< 5	< 5	< 5	< 5	< 5	< 5	< 5	< 5	< 5	< 5
Rb	< 1	1	< 1	< 1	< 1	1	< 1	< 1	1	< 1	< 1
Th	< 1	< 1	1	< 1	< 1	< 1	2	< 1	< 1	< 1	< 1

Harmful elements and economic uses of talc deposits, although S and P are very minor components in all the talc ore types of the area under study and do not affect their industrial use. Cu is not detected at all. Fe and Mn contents are considerably high; therefore, the ore should be treated from the excess of this element to be used as an electrical insulator. As is always less than 5ppm, so the ore can be used in the cosmetic industry without treatment.

### Oxygen and carbon isotopes

The carbon and oxygen isotope study of 4 carbonate-rich samples are presented in Table 8. On the basis of petrographic studies (cathodoluminescence and stained uncovered thin sections), samples containing different types of carbonate minerals were selectively chosen. Results are expressed in standard as per mil notation relative to Pee Dee Belemnite (PDB) and standard mean oceanic water (SMOW). Analytical error is in the range of 0.1–0.2 per mil for both^18^ O and^13^ C the present analyses were carried out at the University of Liverpool Stable Isotope laboratory.

The main applications of oxygen isotope geochemistry in carbonate system (as the most common mineral parentage for talc in the studied areas) are to estimate the temperature of mineral formation and provide information on the origin and chemistry of the hydrothermal fluids.

**Table 8:** Isotopic data of the analyzed samples:

Phases		Magnesite			Dolomite			Calcite		
Kind of isotopes		O^18^		C^13^	O^18^		C^13^	O^18^		C^13^
Calculations To		PDB	SMOW		PDB	SMOW		PDB	SMOW	
G. El- Maiyit	5	–	–	–	−3.77	27.02	−9.36	–	–	–
	6	–	–	–	−1.5	29.36	−4.87	–	–	–
	8	−16.36	14.04	−8.27	–	–	–	–	–	–
	4	−10.07	20.52	−2.97	–	–	–	–	–	–

*PDB* Pee Dee Belemnite, *SMOW* Standard mean oceanic water.

All the ultramafic samples have negative oxygen isotope values. Magnesite analyses of ultramafic samples have lower σO^18^ values (-16.36 to − 10.07) than those from dolomite samples (-3.27 to -1.5). All carbonate - minerals (magnesite and dolomite) show a more or less similar σ^13^C in all analyzed samples. The carbonates are chosen the analysis because they are considered to be the main parentage source of the studied talc deposits.

The obtained results of^13^C for magnesite (−2.97: −8.27%) revealed that the carbon source is the atmospheric CO_2_ (σ^13^C is −7%) which impinge on the fluid surface perhaps coupled with minor amount from the mantle materials (σ^13^Cis – 4 to − 8%). O’Neil and Barnes^69^ reported that, magnesites (the cryptocrystalline varieties) associated with serpentinite and ultramafic rocks show marked^13^C depletion reflecting the isotopic composition of CO_2_ derived from metamorphic reactions at depth.

The obtained σ^13^C SMOW values for ultramafic rocks revealed that the fluids involved during carbonate formation was metamorphic water. Taylor (1979^70^) reported that the values of oxygen isotope for various waters are as; 7.9:9 per mil for magmatic water, similar values for hydrothermal water while metamorphic water has higher values commonly from 10 to 14% (marble has a value of 17: 24%). Oceanic water is uniform at -5:5% and meteoric water has values between − 10 and − 4%. According to the natural oxygen isotope reservoirs^70,71^, the magmatic and/or metamorphic waters rather than meteoric seem likely to be the origin of the hydrothermal fluids which circulated in the system during carbonatization talc-carbonate alteration of ultramafic rocks.

According to Bone^72^, magnesite analyses for σ^18^ O can be used to determine the temperature of the parental fluids. The pure magnesite phase of G. El-Maiyit was formed at a low temperature around 100^o^C while magnesite in talc-carbonate rock was formed at higher temperature (140–175^o^C).

## Conclusions

Our study integrated petrographical, mineralogical, and petrochemical analyses with remote sensing image analysis to investigate the G. El-Maiyit talc deposit and its host rocks, leading to the following conclusions:

1. Remote sensing investigations utilizing Sentinel-2 data revealed a clear distinction of serpentinite rocks and their associated talc deposits. While the FCC effectively illustrated the distribution of these rock bodies, the PC2-PC1-PC3 band combination provided superior discrimination, highlighting the talc deposits in orange with enhanced spatial clarity when integrated with PRISM high-resolution data. Subsequently, detailed mineralogical and geochemical analyses were conducted to evaluate the feasibility of the identified talc deposits.
2. Talc deposit of G.El-Maiyit is present within a belt of almost entirely serpentinized ultramafic rocks of Alpine type characters which are emplaced into metamorphosed sedimentary and volcanic rocks. In a several places, the serpentinite rock is mainly altered to talc-carbonate.
3. Serpentinization process occurred without any obvious loss of the major components such as silica, magnesia, iron, aluminum, chromium and nickel as shown from the chemical data, although these elements were redistributed to new mineral phases. CO_2_, H_2_O and S being added during the alteration. The R O` / SiO ratio ranges from 1.37 to 1.64 and averaged 1.59%. This ratio reflects the variation in the pyroxene and olivine contents in the original parent ultramafic body, which was mainly peridotite. The average MgO/SiO_2_of the analyzed samples range from 0.872 to 1.014 with an average of 0.89 for the more pyroxene-rich harzburgite and 0.98 for olivine-rich harzburgite.
4. Generally, the analyses are depleted in REE relative to the chondrite REE abundance. The pyroxene-rich harzburgite samples have REE abundance controlled by the modal amount and REE content of olivine, orthopyroxene and plagioclase in the original ultramafic body. The REE of the olivine-rich harzburgite samples are dominated by the modal amount of olivine relative to orthopyroxene. Accordingly, the olivine-rich harzburgite is more depleted in REE (ranges between 0.99 and 1.29 ppm) compared to the pyroxene-rich one (3.09-8.48ppm).
5. The parent ultramafic bodies were parts of upper mantle and emplaced on the continental crust after partial melting and formation of olivine, pyroxene and chromite. The emplacement is followed by regional metamorphism and serpentinization, shearing and hydrothermal alteration. The shear surfaces due to the tectonic effects are common in the area. They are act as channels for the circulation of the fluids and subsequently localizing the mineralization.
6. Talc deposit has a consistent mineralogical composition and according to the mineral abundance, three main ore types have been distinguished which are pure talc, tremolite-talc and chlorite-talc ore types. The mineral assemblage of the talc ore is talc, tremolite, chlorite, chrysotile, antigorite and chromite. Pure talc ore type accommodates few percent of impurity minerals (< 10) as tremolite, chlorite and chromite compared to other ore types. This is confirmed chemically, where the summation of talc components (SiO_2_, MgO and H_2_O) decreases from pure talc ore (94.19%) to tremolite-talc (91.76%) and chlorite talc (82.97%).
7. On the thermal curves talc (900–1000^o^C) chlorite (645, 835, 860^o^C) and tremolite 1000–1045^o^C ) show characteristic thermal effects.
8. Chemical composition of talc and its associated minerals in the different ore types are studied by SEM. The substitution elements (Cr, Ni and iron) are known to be enriched in ultramafic rocks unlike volcanic ones. The structural formula of talc Mg_2.931_ Fe ^2+^_0.054_ Fe ^3+^_0.079_ Ni _0.012_ Mn_0.001_ Cr _0.001_Si _3.93_ O _10_ O _10_OH _2_, tremolite is Ca _1.816_ ( Mg _4.611_ Fe ^2+^_0.477_ Fe^3 +^ _0.050_ M n _0.040_Cr _0.012_ Si_7.952_ Al _0.034_ O_22_ (OH, F)_2_, actinolite is Ca _1.798_ Mg _4.447_ Fe^2 +^ _0.529_ Fe^3+^ Mn _0.04_ Cro._050_) Si_7.814_ Al_0.242_ O _22_ (OH, F) _2_ and chlorite is Mg_8.70_ Fe^2 +^ _1.19_ Al^vi^_1.846_ ) (Si _5.987_ Al^1V^_2.013_ ) O_20_ (OH) _16_ From the structural formula, the temperature of the chlorite formation ranges from 164 to 251^o^C with an average of 231^o^C .
9. The major elements chemistry shows that, there is a very little or perhaps no variation in the contents of MgO, Fe_2_O_3,_ FeO, NiO and Cr_2_O_3_ between the talc deposit and its parent rock (serpentinite). Only, silica was added with large extent to Mg-rich rocks.
  *Mg*_*3*_*Si*_*2*_*O*_*5*_*(OH)*_*4*_*+2SiO*_*2*_ *= Mg*_*3*_*SiO*_*10*_*(OH)*_*2*_*+H*_*2*_*O.*
  There is no marked variation in the contents of trace elements between the serpentinite rock and talc deposit. Only, slight depleted in contents of Sr, V and Co (3, 10 and 43 ppm respectively) and slight enriched in Zn (47 ppm) contents are observed in talc deposit.
10. The carbon and oxygen isotopes for the samples from ultramafic rocks are studied and the following results are recorded;
  In terms of source of fluids, the metamorphic and/or magmatic waters are supposed to be the main fluids which are circulated during the hydrothermal alteration.
  The carbon and oxygen contents of magnesite revealed that the pure phase of G. El-Maiyit is formed at around 100 °C. Magnesite contained in serpentinite and talc-carbonate rocks of G. El-Maiyit is formed at higher temperature (140–175 °C).
  The atmosphere is the possible source of carbon in the carbonate formation. The results revealed the possibility of participation of carbon from the mantle materials.
11. In general, the talc deposit associated with the serpentinite belt of G. El-Maiyit is similar to the talc deposit of Vermont which has been shown to formed by metasomatic alteration of ultramafic parentage^73^. The petrographic and petrochemical studies that the serpentinite and associated talc were derived from the same protolith and the variation in the mineralogy and chemical composition is due to the movements of MgO, SiO_2_ and H_2_O during the regional metamorphism.

## Data Availability

The datasets used and/or analyzed during the current study are available from the corresponding author upon reasonable request.
